# Contribution of Mitochondrial Activity to Doxorubicin-Resistance in Osteosarcoma Cells

**DOI:** 10.3390/cancers15051370

**Published:** 2023-02-21

**Authors:** Isabella Giacomini, Margherita Cortini, Mattia Tinazzi, Nicola Baldini, Veronica Cocetta, Eugenio Ragazzi, Sofia Avnet, Monica Montopoli

**Affiliations:** 1 Department of Pharmaceutical and Pharmacological Sciences, University of Padua, 35131 Padova, Italy; 2 Biomedical Science and Technologies and Nanobiotechnology Lab, IRCCS Istituto Ortopedico Rizzoli, 40136 Bologna, Italy; 3 Department of Biomedical and Neuromotor Sciences, Alma Mater Studiorum, University of Bologna, 40126 Bologna, Italy; 4 Veneto Institute of Molecular Medicine (VIMM), 35129 Padova, Italy; 5 Institute of Oncology Research (IOR), Oncology Institute of Southern Switzerland (IOSI), 6500 Bellinzona, Switzerland

**Keywords:** osteosarcoma, cancer, chemotherapy, doxorubicin, drug resistance, metabolism, mitochondria, targeting mitochondrial alterations

## Abstract

**Simple Summary:**

Osteosarcoma represents the most common bone tumor, and it is the second most fatal cancer in children and young adults. However, despite the clinical benefit of chemotherapy, osteosarcoma patients still have a poor prognosis due to the common onset of drug resistance, as well as the disease’s growth and metastasis frequency. Thus, understanding the processes underlying the phenomenon of resistance represents a challenge in the field of oncology and may lead to new approaches to overcome resistance. This study highlights a different mitochondrial phenotype in doxorubicin-resistant cancer cells and suggests targeting the altered pathway to re-sensitize the resistant clones to the drug. Taken together, these results contribute to identifying the mitochondrial metabolism changes and to developing combined approaches with the final goal of overcoming doxorubicin resistance in osteosarcoma.

**Abstract:**

Osteosarcoma is considered the most common bone tumor affecting children and young adults. The standard of care is chemotherapy; however, the onset of drug resistance still jeopardizes osteosarcoma patients, thus making it necessary to conduct a thorough investigation of the possible mechanisms behind this phenomenon. In the last decades, metabolic rewiring of cancer cells has been proposed as a cause of chemotherapy resistance. Our aim was to compare the mitochondrial phenotype of sensitive osteosarcoma cells (HOS and MG-63) versus their clones when continuously exposed to doxorubicin (resistant cells) and identify alterations exploitable for pharmacological approaches to overcome chemotherapy resistance. Compared with sensitive cells, doxorubicin-resistant clones showed sustained viability with less oxygen-dependent metabolisms, and significantly reduced mitochondrial membrane potential, mitochondrial mass, and ROS production. In addition, we found reduced expression of *TFAM* gene generally associated with mitochondrial biogenesis. Finally, combined treatment of resistant osteosarcoma cells with doxorubicin and quercetin, a known inducer of mitochondrial biogenesis, re-sensitizes the doxorubicin effect in resistant cells. Despite further investigations being needed, these results pave the way for the use of mitochondrial inducers as a promising strategy to re-sensitize doxorubicin cytotoxicity in patients who do not respond to therapy or reduce doxorubicin side effects.

## 1. Introduction

Osteosarcoma is a severe bone tumor mostly affecting children and adolescents [1]. Its worldwide incidence rate reaches four cases per million per year [2,3]. Current therapeutic management includes surgery, radiotherapy, and chemotherapy [2]. The standard of care for chemotherapy is represented by methotrexate, cisplatin, and doxorubicin, or a combination of these three agents [4].

Doxorubicin (DXR) is a chemotherapeutic agent intercalating DNA and belongs to the anthracycline family. The mechanism of action consists of the inhibition of DNA topoisomerase II, which blocks DNA replication hampering the recombination of DNA double-strand. Recently, another mode of action for DXR has been proposed: this mechanism involves increased reactive species of oxygen (ROS) levels which causes DNA damage and leads to cell death [5]. The successful use of doxorubicin in clinical therapy has been jeopardized by severe toxicity, such as cardiotoxicity [6], and the onset of drug resistance [7]. 

The main known mechanisms of DXR resistance involve increased drug efflux due to P-glycoprotein expression, which is also associated with a worse outcome [8,9], decreased drug influx as well as drug compartmentalization into lysosomes [10], and enhanced DNA repair or detoxification systems [11]. In the last decades, even the deregulation of energy metabolism has been described as a hallmark of cancer and drug resistance. This phenomenon is also called metabolic reprogramming and it involves several alterations in metabolic pathways, such as increased glycolysis, a shift toward the pentose phosphate pathway, as well as enhanced lipid synthesis, higher exploitation of glutamine pathway, and mitochondrial alterations [12]. Several studies highlighted the central role that mitochondria play in cancer development and progression [13], and in the onset of drug resistance, suggesting that targeting the mitochondrial pathway could represent a promising strategy to overcome drug resistance [7].

Previous studies in our laboratory already identified that mitochondrial alterations are strictly linked with chemotherapy resistance. In particular, we demonstrated that ovarian and osteosarcoma cisplatin-resistant cancer cells presented defects in mitochondrial morphology, in particular reduced mitochondrial mass, suggesting an enhanced activation of mitophagy and a central role played by mitochondria in cisplatin resistance [14,15]. Similarly, in DXR-resistant cells with a multidrug-resistant (MDR) phenotype, we also demonstrated a lower mitochondrial activity [16].

In light of these considerations, our hypothesis is that DXR resistance in osteosarcoma cell models could be driven also by mitochondrial dysfunctions. The purpose of this paper is to characterize the mitochondrial phenotype of two osteosarcoma cell lines, MG63 and HOS, analyzing both sensitive and DXR-resistant clones with the aim of identifying alterations in resistant clones that could be exploited for cancer therapy. 

## 2. Materials and Methods

### 2.1. Cell Lines

The HOS and MG63 cell lines were kindly provided by IRCCS Istituto Ortopedico Rizzoli (IOR, Bologna, Italy), and derived from human osteosarcoma. The cell lines included in our research are commercially available; for this reason, it is not necessary to obtain ethical approval. The DXR-resistant variants HOS DXR 10 ng/mL, HOS DXR 30 ng/mL, HOS DXR 100 ng/mL, as well as MG63 DXR 30 ng/mL and MG63 DXR 100 ng/mL, were obtained by continuous exposure to increasing DXR (Teva Pharmaceutical Industries, Tel Aviv, Israel) concentrations, as reported by Avnet and coworkers [10]. Approximately 2–3 weeks (4–5 passages) were required to establish adequate growth at each DXR concentration. The cells were grown in Iscove’s Modified Dulbecco’s Medium (IMDM) medium (Corning, New York, NY, USA) supplemented with 10% fetal bovine serum (FBS) (Thermo Fisher Scientific, Waltham, MA, USA), 4 mM glutamine (Corning, New York, NY, USA), 100 U/mL penicillin, and 100 µg/mL streptomycin (Corning, New York, NY, USA), in humidified condition at 5% CO_2_ and 37 °C. Cells were collected every 2 days with the minimum amount of 0.25% trypsin-0.2% EDTA (Euroclone, Milan, Italy). 

### 2.2. Mitochondrial Network by Confocal Microscopy

Cells were grown on glass coverslips for 24 h in a complete medium. Then, cells were fixed with paraformaldehyde 4% in phosphate buffer saline solution (PBS), stained with 20 nM Mitotracker Orange (ex: 554 nm/em: 576 nm) (Thermo Fisher Scientific, Waltham, MA, USA) for 30 min and Hoechst 33342 (Thermo Fisher Scientific, Waltham, MA, USA) for 20 min. Images were acquired by confocal microscopy LSM 800 and software ZEN 2.1 60× objective and analyzed by ImageJ version 1.53t (U.S. National Institutes of Health, Bethesda, MA, USA).

### 2.3. Mitochondrial Membrane Potential by Flow Cytometry

Cells were seeded in 12-well plates and grown in complete medium, washed with PBS, detached with 0.25% trypsin-0.2% EDTA and centrifuged for 5 min at 1200 rpm. Cells were stained with rhodamine-123 10 μM (Sigma-Aldrich, St. Louis, MO, USA) and incubated for 15 min. Fluorescence intensity was analyzed using an Epics XL flow cytometer. Mean fluorescence intensity (MFI) values were obtained using the EXPO 32 software. 

### 2.4. Mitochondrial Mass by Flow Cytometry

Cells were seeded in 12-well plates and grown in complete medium, washed with PBS, detached with 0.25% trypsin-0.2% EDTA and centrifuged for 5 min at 1200 rpm. Cells were resuspended with nonyl acridine orange (NAO) 1.25 μM (Sigma-Aldrich, St. Louis, MO, USA) and incubated for 15 min. Fluorescence intensity was analyzed using an Epics XL flow cytometer version 3.0. Mean fluorescence intensity (MFI) values were obtained using the EXPO32 software (Applied Cytometry Systems, Dinnington, Sheffield, UK). 

### 2.5. Quantitative Real-Time PCR

Total mRNA was isolated with TRIzol (Themo Fisher, Waltham, MA, USA) as previously described by Chomczynski and Sacchi [17] and quantified with Nanodrop. The relative expression of each gene was determined by quantitative real-time PCR (Eco™ Illumina, Real-Time PCR system, San Diego, CA, USA) using One Step SYBR PrimeScript RT-PCR Kit (Takara Bio, Otsu, Shiga, Japan) and the primers designed as follow: *PGC-1α*: F 5′-ACACAGTCGCAGTCACAACAC-3′ R 5′-GGAGTGGTGGGTGGAGTTAGG-3′; *NRF1*: F 5′-GCTTGCGTCGTCTGGATGG-3′ R 5′-GTAACCCTGATGGCACTGTCTC-3′; *NRF2*: F 5′-TTCCTTCAGCAGCATCCTCTCC-3′ R 5′-AATCTGTGTTGACTGTGGCATCTG-3′; *TFAM:* F 5′-AACAACGAAAATATGGTGCTGAGG-3′ R 5′-CAAGTATTATGCTGGCAGAAGTCC-3′; *BNIP3*: F 5′-GAATTTCTGAAAGTTTCCTTCCA-3′ R 5′-TTGTCAGACGCCTTCCAATA-3′. Linearity and efficiency of PCR amplifications were assessed using standard curves generated by serial dilution of complementary DNA; melt-curve analysis was used to confirm the specificity of amplification and the absence of primer dimers. All genes were normalized to *CALNEXIN* or *GAPDH* designed as follows: *CALNEXIN*: F 5′-GAAGGGAAGTGGTTGCTGTG-3′; R 5′-GATGAAGGAGGAGCAGTGGT-3′; *GAPDH*: F 5′-AATCCCATCACCATCTTCCA-3′ R 5′-TGGACTCCACGACGTACTCA-3′. Expression levels of the indicated genes were calculated by the ΔΔCt method using, respectively, the dedicated StepOne software version 2.1 (Applied Biosystems, Waltham, MA, USA) or Eco™ Software v4.0.7.0 (Illumina Inc., San Diego, CA, USA). 

### 2.6. Western Blotting

The Lowry method was used to evaluate the protein content after the cells had been seeded in 6-well plates, cultured in complete media, and lysed using ice-cold lysis buffer supplemented with protease inhibitor cocktails (Roche Molecular Biochemicals, Mannheim, Germany) (Biorad DC Protein Assay, MA, USA). In the running buffer, 25 µg of protein from each sample was placed onto a polyacrylamide gel and electrophoretically separated. The proteins were blotted onto a Hybond-P PVDF membrane following electrophoresis (Amersham Biosciences, Buckinghamshire, UK). The membrane was exposed to anti-TOM20 (mouse, 1:1000; AbCam, Cambridge, UK), anti-VDAC1 (mouse, 1:1000; AbCam, Cambridge, UK), and anti-BNIP3 (rabbit, 1:1000; AbCam, Cambridge, UK) after blocking with a 10% skim milk solution. Following washing, the membrane was incubated with HRP-conjugated anti-rabbit secondary antibody (1:3500; PerkinElmer, MA, SUA) or anti-mouse secondary antibody (1:10000; PerkinElmer, MA, USA). According to the manufacturer’s instructions, the signal was seen using an improved chemiluminescent kit from Amersham Biosciences, and then it was examined using a Molecular Imager VersaDoc MP 4000 (Biorad, Hercules, CA, USA). Proteins were normalized to β-ACTIN (mouse, 1:7000, AbCam, Cambridge, UK) and GAPDH (rabbit, 1:2000, Cell Signaling, Danvers, MA, USA). 

### 2.7. Mitochondrial ROS Levels by Flow Cytometry

A constant number of cells were seeded, and after 48 h, 5 μM Mitosox (Invitrogen, Waltham, MA, USA) was added and incubated for 30 min at 37 °C. Cells were then washed in PBS solution, detached using 0.05% trypsin and 0.02% EDTA, centrifuged for 10 min at 2000 rpm, and resuspended in a solution of PBS, 2% FBS, and 0.02% sodium azide (Sigma-Aldrich, St. Louis, MO, USA). Cells were then examined directly using a 488 Argon laser-equipped Epics XL Coulter Systems (Beckman Coulter, Brea, CA, USA). Dead cells were electronically gated out using forward versus side scatter profiles, and a minimum of 10^4^ cells of interest were then examined further. Analysis using the EXPO 32 software produced the percentages of the fluorescent cells’ values (Beckman Coulter, Brea, CA, USA).

### 2.8. Apoptosis Detection by Flow Cytometry

Cells were seeded in 6-well plates and grown in complete medium, washed with PBS, detached with 0.25% trypsin-0.2% EDTA and centrifuged for 5 min at 1200 rpm. Cells were stained with FITC Annexin V (InVitrogen, Waltham, MA, USA) and Propidium Iodide according to manufacturer’s instructions, and then diluted with Annexin V-binding buffer (InVitrogen, Waltham, MA, USA). Fluorescence intensity was analyzed using BD FACS Aria instrument. 

### 2.9. Cell Viability Assays

#### 2.9.1. Crystal Violet Assay

Crystal Violet Assay exploits the ability of this dye to bind the DNA and proteins of adherent cells. For the assay, cells were seeded in 96-well plates, and, following overnight incubation, cells were treated with DXR (1-10-50-100-250-500 ng/mL) and quercetin (0.1-1-5-10-25-50-100-200 μM). After 24, 48, and 72 h of treatment, cells were fixed with paraformaldehyde 4% in PBS. Then, cells were stained with Crystal Violet (Sigma-Aldrich, St. Louis, MO, USA) solution 0.1 % and resuspended in acetic acid 10% in water. The absorbance (Abs) was measured at 590 nm using a Victor3X multilabel plate counter (Wallac Instruments, Turku, Finland). 

#### 2.9.2. Trypan Blue Assay

Cells were plated on 12-well plates and, following overnight incubation, were exposed to different rotenone treatments (0.01–0.1–1 μM) according to the experimental protocol. After treatments, cells were washed, detached with 0.25% trypsin-0.2% EDTA, and suspended in trypan blue (Sigma-Aldrich, St. Louis, MO, USA) at a 1:1 ratio in a medium solution. Cells were counted using a chamber Burker hemocytometer. 

#### 2.9.3. Hoechst Cell Count in Hypoxic Conditions

Cells were plated on 96-well plates and, following overnight incubation, were exposed to 100 μM cobalt chloride (Sigma-Aldrich, St. Louis, MO, USA). 24 and 48 h from incubation with cobalt chloride, cells were stained with Hoechst 33258 2.25 μg/mL (Thermo Fisher Scientific, Waltham, MA, USA) for 20 min. To induce hypoxia from oxygen deprivation, cells were plated on 96-well plates and, following overnight incubation, were moved to the Environmental Chamber of ImageXpress Pico Automated Cell Imaging System (Molecular Devices, San Jose, CA, USA) at 2% O_2_ concentration. For both the chemically induced hypoxia and hypoxia from oxygen deprivation, cells were stained with Hoechst 33258 2.25 μg/mL for 20 min at different time points. Images were acquired by fluorescent microscopy ImageXpress Pico Automated Cell Imaging System with a 10× objective. Analysis software detected the total number of cells based on Hoechst positive nuclei.

### 2.10. Statistical Analysis

The statistical analysis of experimental results was performed using GraphPad Prism version 7 (GraphPad, San Diego, CA, USA.). An appropriate post-hoc test (Bonferroni, Tukey—Kramer, or Šídák multiple comparison tests) was used after the one-way or two-way analysis of variance (ANOVA) of the data. For the purpose of assessing statistical significance, a *p*-value of less than 0.05 was taken into account.

## 3. Results

### 3.1. DXR-Resistant Clones Demonstrate Changes in Mitochondrial Morphology and Loss in Mitochondrial Membrane Potential

Mitochondrial morphology can be modified in response to environmental and cellular stresses, such as DXR treatment. This process is regulated by fusion and fission dynamics and plays a pivotal role in cellular activity [18]. 

**Figure 1:**
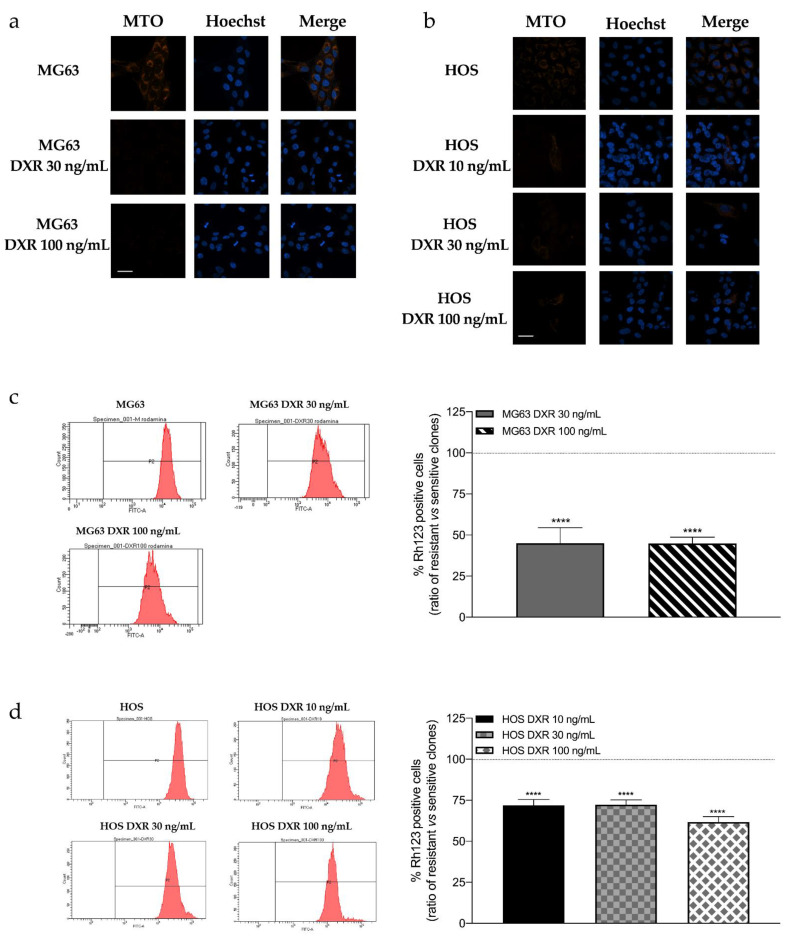
(**a**) Images representing mitochondrial network morphology of sensitive and resistant clones: MG63, MG63 DXR 30 ng/mL, and MG63 DXR 100 ng/mL. (**b**) Images representing mitochondrial network morphology of sensitive and resistant clones: HOS, HOS DXR 10 ng/mL, HOS DXR 30 ng/mL, HOS DXR 100 ng/mL. Mitotracker Orange (ex:554 nm/em:576 nm) was used to stain mitochondria, while Hoechst 33342 was used to stain nuclei. Images were acquired by confocal microscopy LSM 800 and software ZEN 2.1 60× objective. Scale bar = 20 μm. (**c**) Evaluation of mitochondrial membrane potential by flow cytometry. Representative cytograms and graphical quantification showing the percentage of fluorescence intensity of Rhodamine 123 (10 μM) in MG63, MG63 DXR 30 ng/mL, and MG63 DXR 100 ng/mL. The horizontal dotted line refers to normalized untreated cells. (**d**) Evaluation of mitochondrial membrane potential by flow cytometry. Representative cytograms and graphical quantification showing the percentage of fluorescence intensity of Rhodamine 123 (10 μM) in HOS, HOS DXR 10 ng/mL, HOS DXR 30 ng/mL, HOS DXR 100 ng/mL cells. The horizontal dotted line refers to normalized untreated cells. Data represent mean ± SD of three different experiments. **** *p* < 0.0001, resistant vs. sensitive cells.

The evaluation of mitochondrial network of sensitive and resistant osteosarcoma cell lines by confocal microscopy was performed through the orange-fluorescent probe Mitotracker Orange, which stains mitochondria with a membrane potential-dependent accumulation. Figure 1a shows the mitochondrial network of sensitive MG63 and resistant clones, DXR 30 ng/mL, and DXR 100 ng/mL, while Figure 1b refers to HOS and relative DXR-resistant clones, DXR 10 ng/mL, DXR 30 ng/mL, and DXR 100 ng/mL. Images suggested no difference in the mitochondrial network, which is similar and filamentous in both sensitive and resistant cancer cells. However, the membrane potential is lower in resistant clones compared to sensitive cells.

To confirm the data obtained by confocal microscopy, the mitochondrial membrane potential of sensitive and resistant osteosarcoma cells was evaluated in both MG63 and HOS cell lines through flow cytometry analysis. Cells were stained with Rhodamine 123, a fluorescent probe sequestered by active mitochondria in a membrane potential fashion. Figure 1c,d shows that all resistant clones significantly reduced membrane potential compared to sensitive clones, thus confirming results obtained with confocal microscopy images. 

These preliminary results suggest an alteration in the mitochondrial status of DXR-resistant osteosarcoma cells, indicating that further investigations are needed to understand the molecular mechanisms responsible for these changes.

### 3.2. The Mitochondrial Mass Was Lower in DXR-Resistant Clones than in Sensitive Cells: Is Mitochondrial Biogenesis or Mitophagy the Involved Pathway?

**Figure 2:**
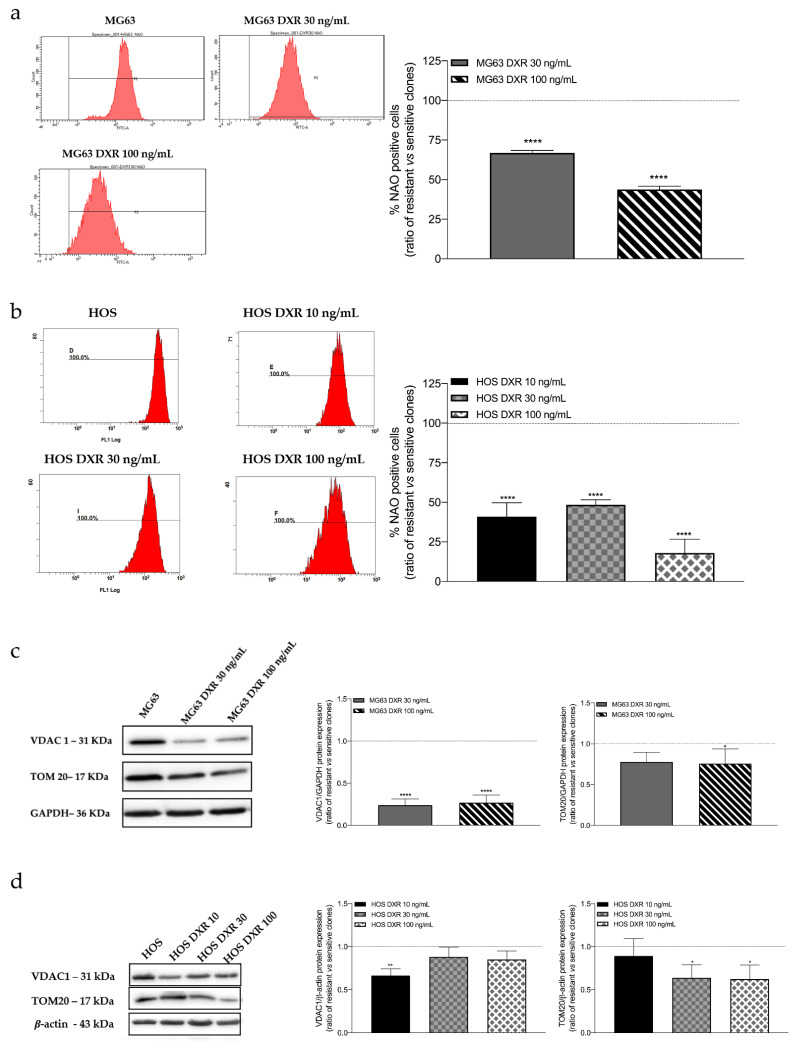
Evaluation of mitochondrial mass by flow cytometry and western blot. (**a**) Representative cytograms and graphical representation showing the percentage of fluorescence intensity of NAO 1.25 μM in MG63, MG63 DXR 30 ng/mL, and MG63 DXR 100 ng/mL cells. The horizontal dotted line refers to normalized untreated cells. (**b**) Representative cytograms and graphical representation showing the percentage of fluorescence intensity of NAO 1.25 μM in HOS, HOS DXR 10 ng/mL, HOS DXR 30 ng/mL, HOS DXR 100 ng/mL cells. The horizontal dotted line refers to normalized untreated cells. Data represent mean ± SD of three different experiments. **** *p* < 0.0001, resistant vs. sensitive cells. (**c**) Representative images and graphical quantification of protein expression of VDAC1 and TOM20, normalized to GAPDH in MG63, MG63 DXR 30 ng/mL, and MG63 DXR 100 ng/mL cells. The horizontal dotted line refers to normalized untreated cells. (**d**) Representative images and graphical quantification of protein expression of VDAC1 and TOM20, normalized to β-actin in HOS, HOS DXR 10 ng/mL, HOS DXR 30 ng/mL, HOS DXR 100 ng/mL cells. The horizontal dotted line refers to normalized untreated cells. The uncropped Western Blot images can be found in Figure S2. Data represent mean ± SD of four different experiments. * *p* < 0.05, ** *p* < 0.01, **** *p* < 0.0001, resistant vs. sensitive cells.

Mitochondria are dynamic organelles and changes in mitochondrial mass reflect unbalance between mitochondrial biogenesis and mitophagy [19]. Increased or decreased mitochondrial mass has been correlated with cancer [20]. Nonyl acridine orange (NAO), a fluorescent probe binding cardiolipin, was used to assess the mitochondrial mass of sensitive and DXR osteosarcoma cells by flow cytometry. Results showed a significant decrease of NAO fluorescence percentage in all resistant clones compared to sensitive cancer cells (Figure 2a,b).

To confirm these data, we even assessed the protein expression of VDAC1 and TOM20, which are already considered specific markers of mitochondrial mass. As it is possible to observe in Figure 2c,d, the expression of both proteins is significantly decreased in resistant cells with respect to their sensitive counterparts. The uncropped Western Blot images can be found in Figures S1 and S2. 

**Figure 3:**
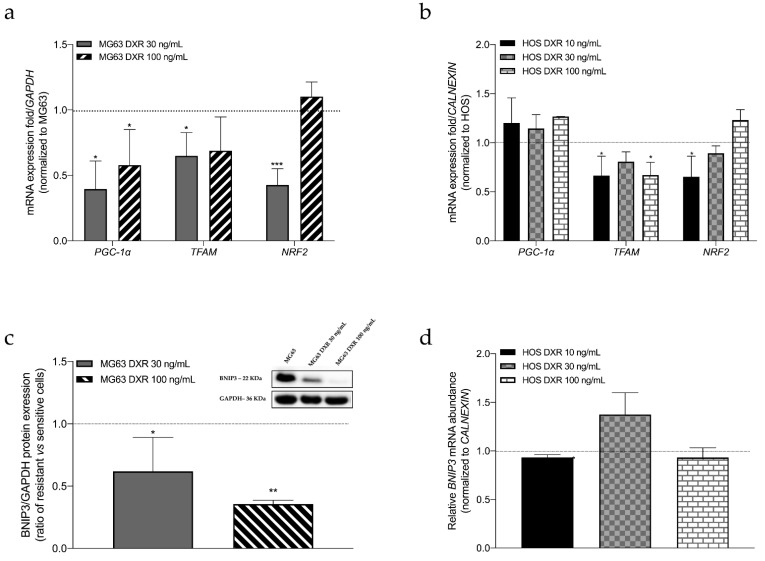
(**a**) Relative mRNA expression levels of genes related to mitochondrial biogenesis in MG63, MG63 DXR 30 ng/mL, and MG63 DXR 100 ng/mL cells. mRNA levels of all genes are normalized to *GAPDH*. The horizontal dotted line refers to normalized untreated cells. (**b**) Relative mRNA expression levels of genes related to mitochondrial biogenesis in HOS, HOS DXR 10 ng/mL, HOS DXR 30 ng/mL, HOS DXR 100 ng/mL cells. mRNA levels of all genes are normalized to *CALNEXIN*. The horizontal dotted line refers to normalized untreated cells. (**c**) Representative images and graphical quantification of protein expression of BNIP3, normalized to GAPDH in MG63, MG63 DXR 30 ng/mL, and MG63 DXR 100 ng/mL cells. The horizontal dotted line refers to normalized untreated cells. The uncropped Western Blot images can be found in Figure S3. (**d**) Relative mRNA expression levels of *BNIP3* in HOS, HOS DXR 10 ng/mL, HOS DXR 30 ng/mL, HOS DXR 100 ng/mL cells. mRNA levels of all genes are normalized to *CALNEXIN*. The horizontal dotted line refers to normalized untreated cells. Data represent media ± SD of three different experiments. * *p* < 0.05, ** *p* < 0.01, *** *p* < 0.001, resistant vs. sensitive cells.

Considering that the equilibrium between mitochondrial biogenesis and mitophagy regulates mitochondrial mass [21], we analyzed some genes involved in both these pathways to dissect which is the process involved in mitochondrial alteration. For mitochondrial biogenesis, we checked the mRNA levels of *PGC1-*α, *TFAM,* and *NRF-2*. As Figure 3a,b shows, the expression of both *PGC1-*α and *TFAM* decreases in MG63 DXR-resistant cells, and *TFAM* levels are lower in HOS DXR-resistant clones. These results suggest that reduced biogenesis could be responsible for the observed reduction of mitochondrial mass in resistant clones. As further confirmation, the protein expression on BNIP3, a marker of mitophagy [22], downregulated in the MG63-DRX resistant clones, thus excluding the involvement of mitophagy as the process responsible for reduced mitochondrial mass (Figure 3c). The uncropped Western Blot images can be found in Figure S2. 

Furthermore, we evaluated the mRNA levels of *BNIP3* in HOS cell lines, as reported in Figure 3d. The results showed that only HOS DXR 30 ng/mL cancer cells slightly increase the expression, but not in a significant way. 

### 3.3. DXR Osteosarcoma Cells Show Decreased mtROS Levels and Different Response to Hypoxia

**Figure 4:**
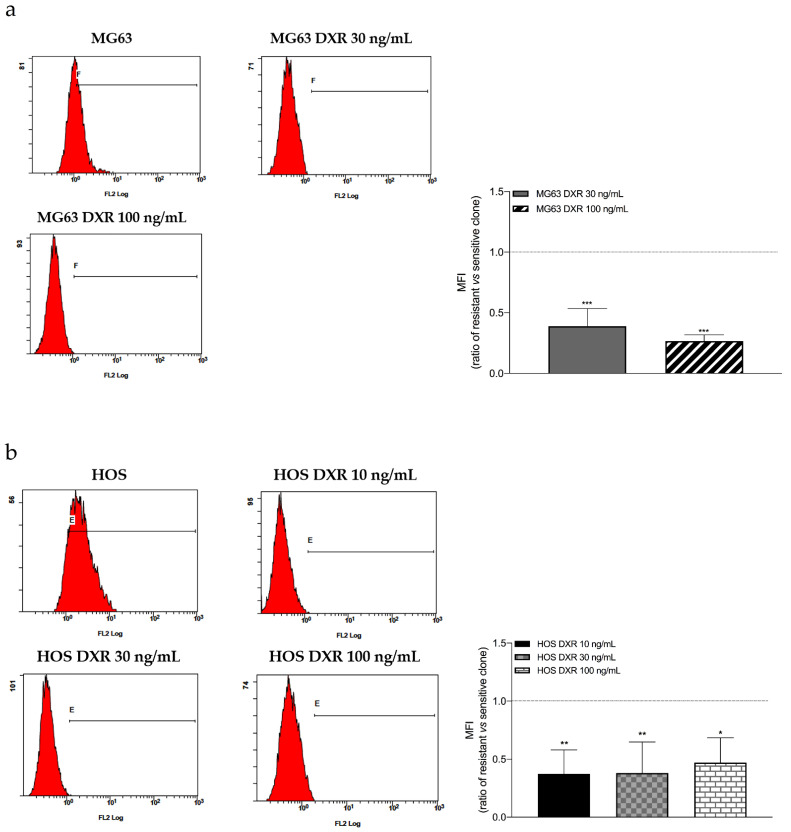
(**a**) Evaluation of ROS levels by flow cytometry. Representative cytograms and relative quantification showing the fluorescence intensity of Mitosox 5 μM in MG63, MG63 DXR 30 ng/mL, and MG63 DXR 100 ng/mL cells. The horizontal dotted line refers to normalized untreated cells. (**b**) Evaluation of ROS levels by flow cytometry. Representative cytograms and relative quantification showing the fluorescence intensity of Mitosox 5 μM in HOS, HOS DXR 10 ng/mL, HOS DXR 30 ng/mL, HOS DXR 100 ng/mL cells. The horizontal dotted line refers to normalized untreated cells. Data represent media ± SD of three different experiments. * *p* < 0.05, ** *p* < 0.01, *** *p* < 0.001, resistant vs. sensitive clones.

Cellular homeostasis is also regulated by the oxidative status, and alterations such as an increase in ROS production represent a factor involved in cell death. In the context of chemotherapy, it is known that the mode of action of several chemotherapeutic agents, such as cisplatin and DXR, is related to oxidative stress induction, which leads to cell death. On the other side, one of the mechanisms of drug resistance is the increased antioxidant system, which counteracts the enhanced chemotherapy-induced oxidative stress [23], and that might be obtained by reducing the biogenesis ROS-producing mitochondria. Therefore, we analyzed the ROS content. As confirmation, results in Figure 4a,b showed ROS decrease in DXR-resistant osteosarcoma cells compared to sensitive counterparts. 

**Figure 5:**
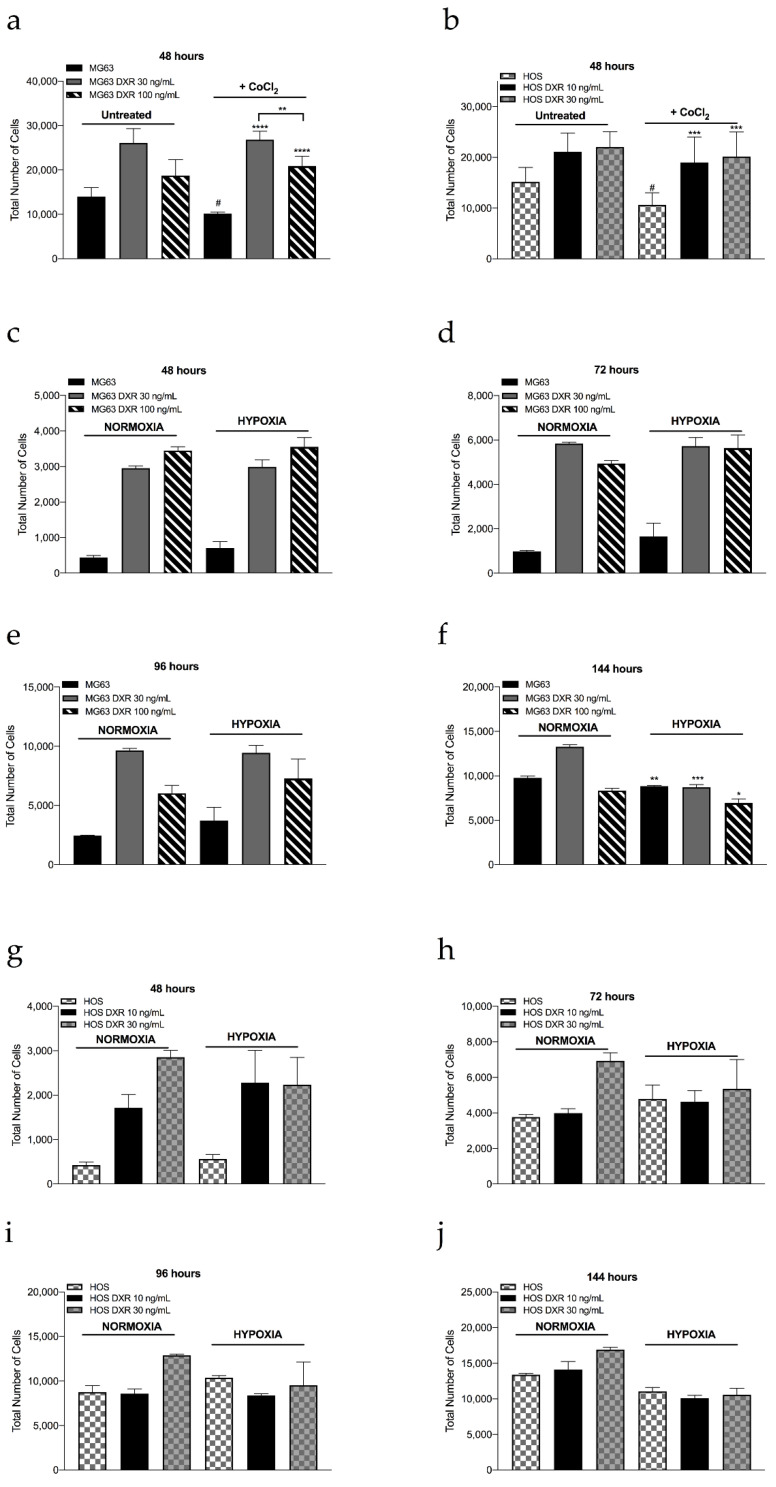
Analysis of cell growth of sensitive and resistant clones under hypoxia for various exposure times. We counted the total number of MG63, MG63 DXR 30 ng/mL, and MG63 DXR 100 ng/mL cells (**a**,**c**–**f**) and HOS, HOS DXR 10 ng/mL, and HOS DXR 30 ng/mL cells (**b**,**g**–**j**) after CoCl_2_ (**a**,**b**) low O_2_ tension (2%) (**c**–**j**) treatments by Hoechst 33258 staining with automated cell count system and at different time points, 48 (**a**–**e**), 72 (**b**–**f**), 96 (**c**–**g**), and 144 h (**d–h**). Data represent mean ± SD of four-eight different experiments. * *p* < 0.05, ** *p* < 0.01, *** *p* < 0.001, hypoxia vs. normoxia conditions; ^#^ *p* < 0.05, CoCl_2_ vs. untreated conditions, *** *p* < 0.001, **** *p* < 0.0001, resistant vs. sensitive clones after CoCl_2_ treatment.

**Figure A1:**
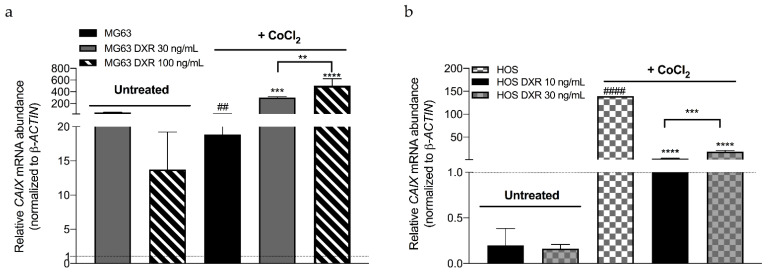
(**a**) Relative mRNA expression levels of *CAIX* gene in MG63, MG63 DXR 30 ng/mL, and MG63 DXR 100 ng/mL cells following treatment with CoCl_2_. mRNA levels of *CAIX* are normalized to β-*ACTIN*. The horizontal dotted line refers to normalized untreated cells. Data represent mean ± SD of four different experiments. ^##^ *p* < 0.01, treated vs. untreated sensitive cells. ** *p* < 0.01, *** *p* < 0.001, **** *p* < 0.0001 treated vs. untreated resistant cells. (**b**) Relative mRNA expression levels of *CAIX* gene in HOS, HOS DXR 10 ng/mL, and HOS3 DXR 30 ng/mL cells following treatment with CoCl_2_. mRNA levels of *CAIX* are normalized to β-*ACTIN*. The horizontal dotted line refers to normalized untreated cells. Data represent mean ± SD of four different experiments. ^####^ *p* < 0.0001, treated vs. untreated sensitive cells. *** *p* < 0.001, **** *p* < 0.0001 treated vs. untreated resistant cells.

**Figure A2:**
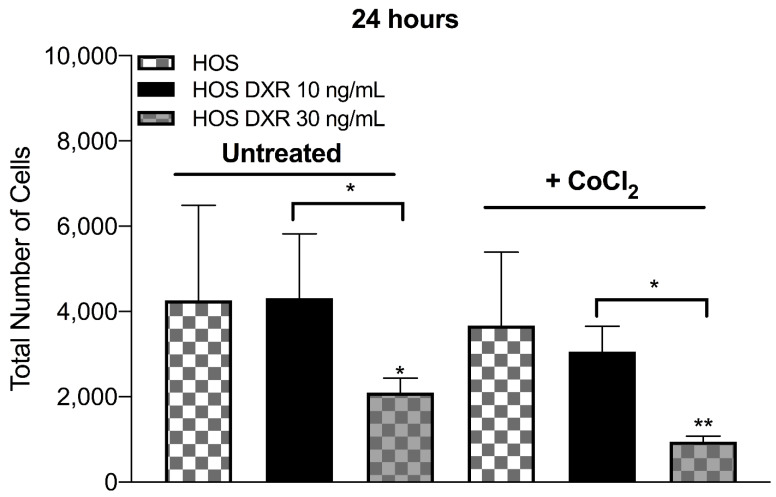
Analysis of cell growth of sensitive and resistant clones under hypoxia for various exposure times. We counted the total number of HOS, HOS DXR 10 ng/mL, and HOS DXR 30 ng/mL cells after CoCl_2_ (**a**,**b**) treatments by Hoechst staining with an automated cell count system and at 24 h. Data represent mean ± SD of four different experiments. * *p* < 0.05, ** *p* < 0.01, resistant vs. sensitive clones after CoCl_2_ treatment.

The reduced mitochondrial mass and biogenesis and the reduced ROS production in resistant cells suggest that these cells have reduced mitochondrial oxidative phosphorylation. To validate this hypothesis, we measured the cellular response to low-oxygen (2%) or chemically induced hypoxia (with CoCl_2_). CoCl_2_ stabilizes hypoxia-inducible factors 1α and 2α under normoxic conditions and simulates a hypoxic microenvironment [24]. As confirmation of the hypoxia-like metabolic state in CoCl_2_-treated cells, mRNA expression levels of the indirect hypoxia marker CAIX [25] were induced in all cells tested (Figure A1). We then counted the number of cells after incubation with both low oxygen tension and CoCl_2_. Before 144 h, cell growth was mostly unaffected by both types of hypoxia, with the exception of sensitive cells treated with CoCl_2_ (Figure A2). In contrast, after longer exposure (at 144 h under low O_2_ tension), all cell types were significantly affected compared with the normoxic condition (Figure 5c–j). Overall, it can be concluded that resistant cells appear to be much less dependent on oxygen metabolism than sensitive cells, as attested by both CAIX expression levels and cell numbers at 48 h when treated with CoCl_2_ (Figure 5a,b).

### 3.4. DXR-Resistant Osteosarcoma Cells Show Different Behavior under Metabolic Stresses

**Figure 6:**
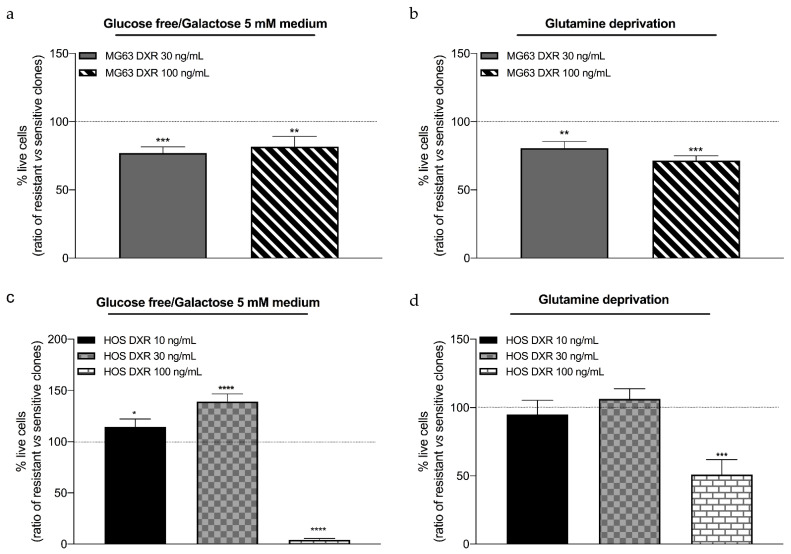
(**a**,**b**) Evaluation of cell viability by Trypan blue assay in MG63, MG63 DXR 30 ng/mL and MG63 DXR 100 ng/mL cells after 24 h in glucose-free/5 mM galactose medium (**a**) and glutamine deprivation (**b**). The horizontal dotted line refers to normalized untreated cells. (**c**,**d**) Evaluation of cell viability by Trypan blue assay in HOS, HOS DXR 10 ng/mL, HOS DXR 30 ng/mL, and HOS DXR 100 ng/mL cells after 24 h in glucose-free/5 mM galactose medium (**c**) and glutamine deprivation (**d**). The horizontal dotted line refers to normalized untreated cells. Data represent mean ± SD of four different experiments. * *p* < 0.05, ** *p* < 0.01, *** *p* < 0.001, **** *p* < 0.0001 resistant vs. sensitive cells.

DXR-resistant osteosarcoma cells revealed a different mitochondrial profile with respect to their sensitive counterpart. Accumulating evidence showed that mitochondrial dysfunctions can be highlighted when oxidative metabolism is increased, such as glucose deprivation and galactose addition in the medium. The next step of this work was to evaluate the response of sensitive and DXR-resistant cancer cells under two metabolic stresses, glucose, and glutamine deprivation. MG63 DXR-resistant clones significantly reduced cell viability after 24 h in both glucose-free/galactose 5 mM, and glutamine-free media (Figure 6a,b). On the other side, among the resistant HOS clones, only HOS DXR 100 ng/mL cells resulted to be affected by these metabolic stresses (Figure 6c,d).

These results, taken together, suggest mitochondrial dysfunctions in DXR-resistant cancer cells, which are more dependent on glucose and glutamine pathways with respect to sensitive counterparts. 

### 3.5. Targeting Mitochondrial Biogenesis to Restore DXR Sensitivity 

**Figure 7:**
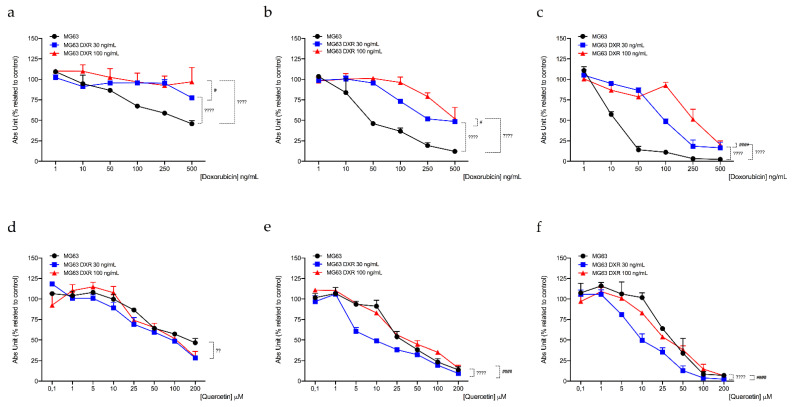
Evaluation of cell viability in MG63, MG63 DXR 30 ng/mL, and MG63 DXR 100 ng/mL following treatment with DXR (**a**–**c**) and quercetin combined with DXR 30 and 100 ng/mL, respectively (**d**–**f**) for 24 (**a**–**d**), 48 (**b**–**e**), and 72 h (**c**–**f**). Data represent mean ± SD of three different experiments. ** *p* < 0.01, **** *p* < 0.0001, resistant vs. sensitive cells; ^#^ *p* < 0.05, ^####^ *p* < 0.0001, DXR100 vs. DXR 30 clones.

**Figure 8:**
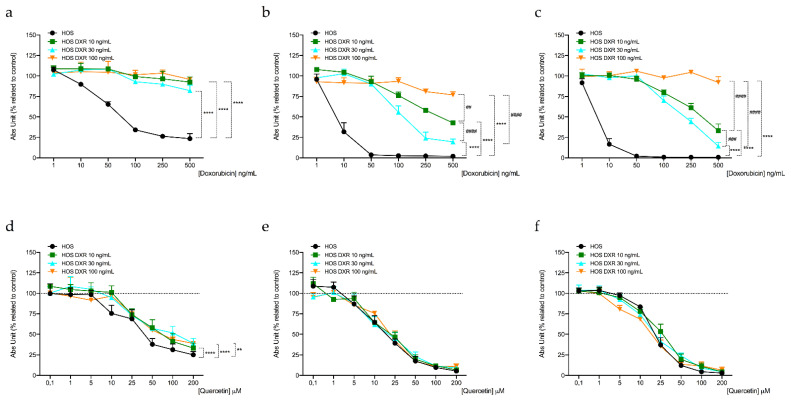
Evaluation of cell viability in HOS, HOS DXR 10 ng/mL, HOS DXR 30 ng/mL, and HOS DXR 100 ng/mL following treatment with DXR (**a**–**c**) and quercetin combined with DXR 10, 30 and 100 ng/mL, respectively, (**d**–**f**) for 24 (**a**–**d**), 48 (**b**–**e**), and 72 h (**c**–**f**). Data represent mean ± SD of three different experiments. ** *p* < 0.01, **** *p* < 0.0001, resistant vs. sensitive cells; ^##^ *p* < 0.01, ^###^ *p* < 0.001, ^####^ *p* < 0.0001, DXR 100 vs. DXR 30 clones, or DXR 30 vs. DXR 10, or DXR 100 vs. DXR 10 clones.

**Figure A3:**
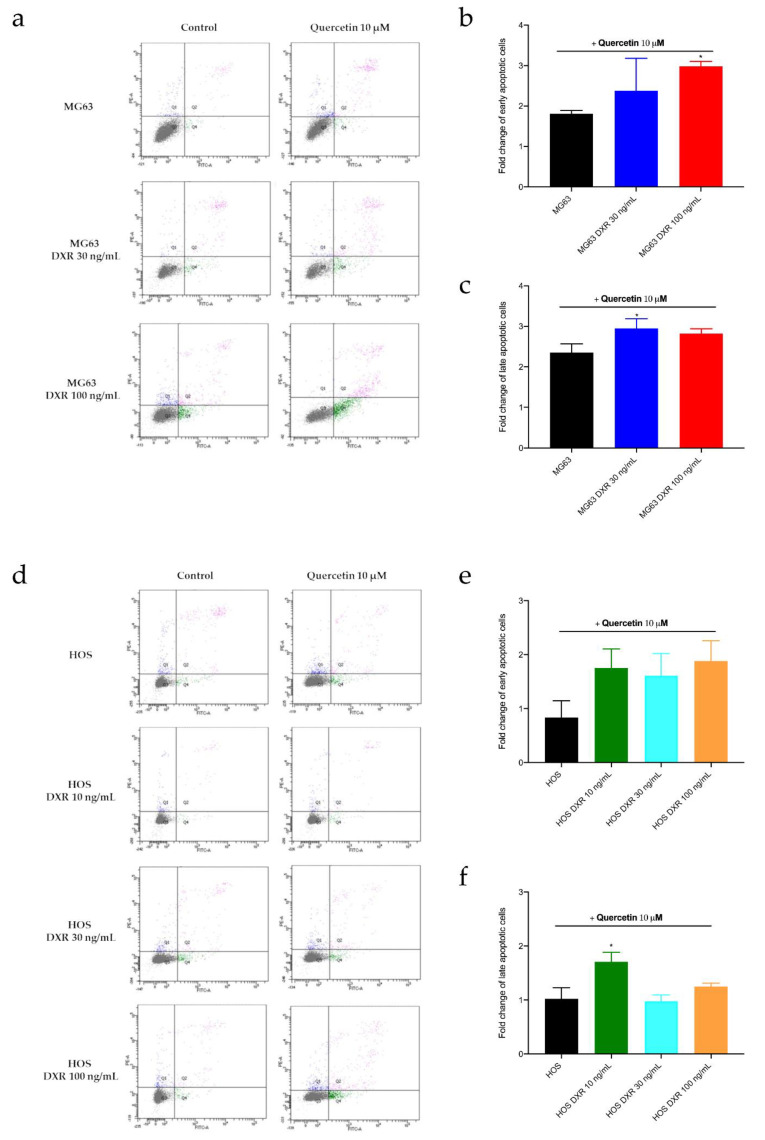
(**a**) Representative cytograms of apoptotic cells analyzed with Annexin V-PI staining. MG63, MG63 DXR 30 ng/mL and MG63 DXR 100 ng/mL cells were treated 72 h with quercetin 10 μM. The graphs represent the quantification of early (**b**) and late apoptosis (**c**) by showing the ratio between treated and untreated. (**d**) Representative cytograms of apoptotic cells analyzed with Annexin V-PI staining. HOS, HOS DXR 10 ng/mL, HOS DXR 30 ng/mL, and HOS DXR 100 ng/mL cells were treated 72 h with quercetin 10 μM. The graphs represent the quantification of early (**e**) and late apoptosis (**f**) by showing the ratio between treated and untreated. Data are shown as mean ± SD of three different experiments. * *p* < 0.05, treated vs. untreated cells.

**Figure A4:**
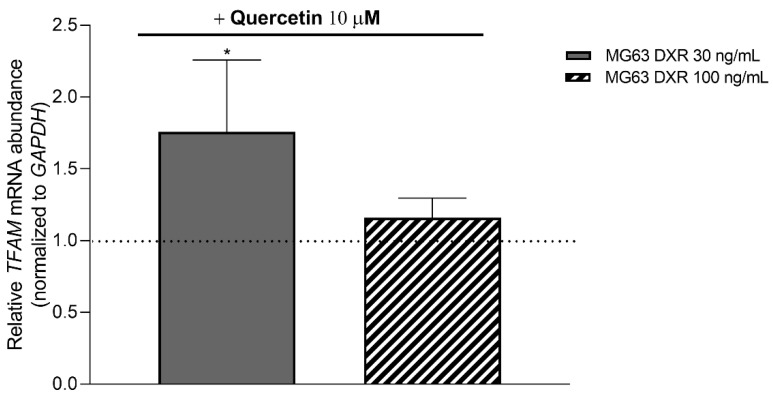
Relative mRNA expression levels of *TFAM* gene in MG63, MG63 DXR 30 ng/mL, and MG63 DXR 100 ng/mL cells following 72 h of treatment with quercetin 10 μM. mRNA levels of *TFAM* are normalized to *GAPDH*. The horizontal dotted line refers to normalized untreated cells. Data represent mean ± SD of three different experiments. * *p* < 0.05, treated vs. untreated cells.

Our results highlighted a different mitochondrial profile of DXR-resistant osteosarcoma cells compared to sensitive cells, in particular demonstrating reduced mitochondrial membrane mass, potential, and activity. Further investigations assessed that the process responsible for decreased mitochondrial mass in resistant cells is likely the reduction of mitochondrial biogenesis. In line with these observations, we reasoned to verify whether the induction of mitochondrial biogenesis was able to re-sensitize DXR effect in osteosarcoma-resistant clones. The previous literature reported that the natural compound quercetin is an inducer of mitochondrial biogenesis [26]. We evaluated cell viability of both sensitive and DXR-resistant cancer cells following 24, 48, and 72 h of the combined treatment with quercetin 0.1–200 μM and DXR, at the respective concentration of each resistant cancer cell lines (DXR 10–30–100 ng/mL). Results showed that the association with quercetin re-sensitized the effect of DXR in MG63 and HOS DXR-resistant cancer cells (Figure 7 and Figure 8, Table 1, Table 2, Tables S1 and S2), by enhancing the percentage of late apoptotic cells (Figure A3). In addition, we also demonstrated that quercetin 10 μM enhanced *TFAM* expression following 72 h of treatment (Figure A4).

These preliminary data suggested that the stimulation of mitochondria biogenesis could improve DXR efficacy in resistant cells; therefore, the combined treatment between mitochondrial biogenesis inducers and the chemotherapeutic drug represents a promising pharmacological approach to overcoming drug resistance. However, further studies will be necessary to identify a selective inducer of this pathway.

## 4. Discussion

Mitochondria play a pivotal role in cell life, coordinating several signaling processes such as the tricarboxylic acid (TCA) cycle, fatty acid oxidation (FAO), and oxidative phosphorylation (OXPHOS) [27], and regulating energy homeostasis and cell death via apoptosis. Recent studies correlated mitochondria and cancer, suggesting that mitochondrial dysfunctions are responsible for tumor development and metastasis progression and also for alterations in chemotherapy’s response. Evidence reported that several chemotherapeutic agents accumulate into mitochondria, thus causing mtDNA damage [28]. These organelles are considered one of the main targets of DXR, through abnormalities in mitochondrial shape and functionality and mitochondrial death mediated by apoptosis. 

Considering the pivotal role of mitochondria in tumor development and in DXR pharmacological activity, this work aimed to characterize the mitochondrial phenotype of sensitive and DXR-resistant osteosarcoma cells. Once alterations were identified, the goal was to target them to restore drug sensitivity in resistant cells. 

Several studies reported that alterations in the mitochondrial network, membrane potential, and mass are correlated with chemoresistance or cancer aggressiveness in different types of tumors such as colon, breast, and gastric cancers [29,30,31]. These aspects reflect the functional and healthy state of these organelles and could become indicators of an unhealthy and dysfunctional status. Our results showed that DXR-resistant cancer cells decreased both the mitochondrial number and the membrane potential, although their morphology remains unchanged. The mitochondrial membrane potential (ΔΨ_m_) is a thorough indicator of mitochondrial function, and its dysregulation causes an increase in reactive oxygen species production, with consequent oxidative damage to DNA and other cellular structures, and ultimately results in cell death [32,33]. It is interesting to note that several cell models have shown a decrease in mitochondrial membrane potential after exposure to DXR in vitro [34], and our data are in line with these findings. In fact, all DXR-resistant osteosarcoma cells showed a reduced mitochondrial membrane potential with respect to the sensitive counterpart. 

It is known that DXR is a redox-active substance that can interact with a number of enzymes, such as mitochondrial complex I, to produce large quantities of ROS and the semiquinone radical [35]. Our results indicate that mitochondria of resistant cells present lower levels of mitochondrial ROS production. Furthermore, DXR-resistant osteosarcoma cells also showed a reduction of mitochondrial mass, confirmed both with NAO fluorescence and the decreased expression of two mitochondrial markers, VDAC1 and TOM20. It is known that mitochondrial mass is regulated by the equilibrium between mitochondrial biogenesis and mitophagy. Alterations of these processes have been correlated with chemoresistance. Peroxisome proliferator-activated receptor-coactivator 1 (PGC-1) and mitochondrial transcription factor A (TFAM), two mitochondrial transcription factors, mediate an increase in mitochondrial number in response to cellular injury [36]. According to several studies, the mitochondrial biogenesis pathway’s genes and proteins are upregulated, and these changes cause cancer chemoresistance. Increased expression of TFAM and PGC-1α was assessed in tumors unresponsive to cisplatin and sorafenib; it was also demonstrated that the depletion of these proteins restored the sensitivity to the drugs [37,38,39]. On the other side, a recent study in our laboratory demonstrated that the increased expression of BNIP3, which is a protein involved in the mitophagy process, is related to cisplatin resistance. Both genetic and pharmacological approaches through inhibition showed a re-sensitization of resistant cancer cells to the chemotherapeutic agent, supporting the idea that targeting mitochondrial dynamics could be a valuable strategy in chemosensitization. Interestingly, our data demonstrated that DXR-resistant osteosarcoma cells decreased mRNA levels of *TFAM* gene compared to their sensitive counterpart, thus suggesting that the downregulation of mitochondrial biogenesis could be the process involved in the DXR resistance onset. In line with this, the evaluation of the mitophagic marker BNIP3 shows that resistant cells didn’t present significant alterations, prompting us to exclude an increased mitophagy as the mechanism responsible for a reduced mitochondrial mass. This result is not very surprising because in previous studies, we have already observed that osteosarcoma cells do not tend to activate autophagy as an adaptation mechanism to stressful conditions [10,40]. 

Having identified the downregulation of mitochondrial biogenesis genes as a possible mechanism of resistance, we sought to combine DXR treatment with a drug that may induce the activation of this process and thus re-sensitize DXR-resistant cells. Quercetin is a natural flavonoid already known to be an inducer of mitochondrial biogenesis [41]. In our DXR-resistant cell model, combining DXR with quercetin re-sensitized the resistant cells to DXR. However, quercetin is also a monocarboxylate transporter (MCT) inhibitor [42]. Since MCTs play a significant role in maintaining intracellular pH by transporting excess protons outside cancer cells, it is also possible that quercetin treatment acidifies the cytosol in resistant cells. The subsequent increase of the pH gradient at the plasma membrane may facilitate the cellular intake of DXR, which is a weakly basic molecule, and thus increase its intracellular accumulation and toxicity [10]. However, in this study, we also showed that quercetin treatment increases *TFAM* levels in MG63-resistant cells, confirming that, in these cells, quercetin treatment has the potential to restore DXR sensitivity by inducing mitochondrial biogenesis. In conclusion, our preliminary results pave the way for *TFAM* inducers or other mitochondrial inducers as possible DXR sensitizers. The combination of compounds acting on different targets represents a promising strategy in the context of chemoresistance therapy because it allows for a reduction in the dosage of single drugs, thus alleviating severe toxicities and at the same time enhancing the therapeutic effectiveness. 

## 5. Conclusions

Taken together, these data highlighted a different mitochondrial profile between sensitive and DXR-resistant osteosarcoma cells. In particular, resistant clones showed a decreased mitochondrial mass and reduced expression of mitochondrial biogenesis genes. The phenotyping of the mitochondrial profile allows the identification of altered pathways, which can be exploited by novel pharmacological approaches that selectively target mitochondrial dysfunctions in resistant cancer cells. In this work, the treatment of osteosarcoma cells with quercetin, a natural compound able to induce mitochondrial biogenesis, caused a re-sensitization of DXR-resistant cells to the chemotherapeutic agent. These preliminary results open the window to the development of promising pharmacological strategies to overcome DXR resistance in osteosarcoma. 

## Figures and Tables

**Table 1:** Cytotoxic effect of quercetin (calculated as IC_50_) after 24, 48, and 72 h of treatment in MG63, MG63 DXR30 ng/mL, and MG63 DXR100 ng/mL cell lines.

Cell Line	IC_50_ Quercetin (μM)^a^		
	24 h	48 h	72 h
**MG63**	124.87 ± 9.03	68.42 ± 4.53	64.71 ± 3.87
**MG63 DXR30 ng/mL**	110.87 ± 2.46	47.03 ± 8.13 *	35.70 ± 2.34 **
**MG63 DXR100 ng/mL**	100.62 ± 7.50 °	84.41 ± 7.54	56.84 ± 8.60

^a^ Values were determined by regression analysis and are expressed as mean ± SD of three different experiments. * *p* < 0.05, ** *p* < 0.01, MG63 DXR30 ng/mL vs. MG63 cells; ° *p* < 0.05, MG63 DXR100 ng/mL vs. MG63 cells.

**Table 2:** Cytotoxic effect of quercetin (calculated as IC_50_) after 24, 48, and 72 h of treatment in HOS, HOS DXR 10 ng/mL, HOS DXR 30 ng/mL, and HOS DXR 100 ng/mL cell lines.

Cell Line	IC_50_ Quercetin (μM) ^a^		
	24 h	48 h	72 h
**HOS**	339.23 ± 6.70	226.80 ± 10.07	224.24 ± 4.72
**HOS DXR10 ng/mL**	536.25 ± 11.46 ^****^	265.30 ± 0.95 ^***^	286.04 ± 1.00 ^****^
**HOS DXR30 ng/mL**	458.51 ± 3.02 °°°°	270.48 ± 3.57 °°°°	262.12 ± 3.39 °°°°
**HOS DXR100 ng/mL**	792.65 ± 7.36 ^####^	236.77 ± 4.16	248.01 ± 2.88 ^####^

^a^ Values were determined by regression analysis and are expressed as mean ± SD of three different experiments. *** *p* < 0.001, **** *p* < 0.0001, HOS DXR 10 ng/mL vs. HOS cells; °°°° *p* < 0.0001, HOS DXR 30 ng/mL vs. HOS cells; ^####^ *p* < 0.0001, HOS DXR 100 ng/mL vs. HOS cells.

## Data Availability

The data presented in this study is available within the article and the Supplementary Materials file.

## Supplementary Materials

The following supporting information can be downloaded at: https://www.mdpi.com/article/10.3390/cancers15051370/s1, Figure S1: Western blot HOS; Figure S2: Western blot MG63; Table S1: Cytotoxic effect of doxorubicin in MG63 cell lines; Table S2: Cytotoxic effect of doxorubicin in HOS cell lines.

## References

[1] Tobeiha M., Rajabi A., Raisi A., Mohajeri M., Yazdi S.M., Davoodvandi A., Aslanbeigi F., Vaziri M., Hamblin M.R., Mirzaei H. (2021). Potential of natural products in osteosarcoma treatment: Focus on Molecular Mechanisms. Biomed. Pharmacother..

[2] Marchandet L., Lallier M., Charrier C., Baud’huin M., Ory B., Lamoureux F. (2021). Mechanisms of resistance to conventional therapies for osteosarcoma. Cancers.

[3] Barani M., Mukhtar M., Rahdar A., Sargazi S., Pandey S., Kang M. (2021). Recent advances in nanotechnology—based diagnosis and treatments of human osteosarcoma. Biosensors.

[4] Argenziano M., Tortora C., Pota E., Di Paola A., Di Martino M., Di Leva C., Di Pinto D., Rossi F. (2021). Osteosarcoma in children: Not only chemotherapy. Pharmaceuticals.

[5] Taymaz-Nikerel H., Karabekmez M.E., Eraslan S., Kırdar B. (2018). Doxorubicin induces an extensive transcriptional and metabolic rewiring in yeast cells. Sci. Rep..

[6] Thorn C.F., Oshiro C., Marsh S., Hernandez-Boussard T., McLeod H., Klein T.E., Altman R.B. (2011). Doxorubicin pathways: Pharmacodynamics and adverse effects. Pharm. Genom..

[7] Guerra F., Arbini A.A., Moro L. (2017). Mitochondria and cancer chemoresistance. Biochim. Biophys. Acta (BBA) Bioenerg..

[8] Baldini N. (1997). Multidrug resistance—A multiplex phenomenon. Nat. Med..

[9] Baldini N., Scotlandi K., Barbanti-Bròdano G., Manara M.C., Maurici D., Bacci G., Bertoni F., Picci P., Sottili S., Campanacci M. (1995). Expression of p-glycoprotein in high-grade osteosarcomas in relation to clinical outcome. N. Engl. J. Med..

[10] Avnet S., Lemma S., Cortini M., Pellegrini P., Perut F., Zini N., Kusuzaki K., Chano T., Grisendi G., Dominici M. (2016). Altered pH gradient at the plasma membrane of osteosarcoma cells is a key mechanism of drug resistance. Oncotarget.

[11] Ha J.S., Byun J., Ahn D.-R. (2016). Overcoming Doxorubicin Resistance of Cancer Cells by Cas9-Mediated Gene Disruption. Sci. Rep..

[12] Morandi A., Indraccolo S. (2017). Linking metabolic reprogramming to therapy resistance in cancer. Biochim. Biophys. Acta (BBA) Rev. Cancer.

[13] Wallace D.C. (2012). Mitochondria and cancer. Nat. Rev. Cancer.

[14] Catanzaro D., Gaude E., Orso G., Giordano C., Guzzo G., Rasola A., Ragazzi E., Caparrotta L., Frezza C., Montopoli M. (2015). Inhibition of glucose-6-phosphate dehydrogenase sensitizes cisplatin-resistant cells to death. Oncotarget.

[15] Vianello C., Cocetta V., Catanzaro D., Dorn G.W., De Milito A., Rizzolio F., Canzonieri V., Cecchin E., Roncato R., Toffoli G. (2022). Cisplatin resistance can be curtailed by blunting bnip3-mediated mitochondrial autophagy. Cell Death Dis..

[16] Fotia C., Avnet S., Kusuzaki K., Roncuzzi L., Baldini N. (2015). Acridine orange is an effective anti-cancer drug that affects mitochondrial function in osteosarcoma cells. Curr. Pharm. Des..

[17] Chomczynski P., Sacchi N. (1987). Single-step method of RNA isolation by acid guanidinium thiocyanate-phenol-chloroform extraction. Anal. Biochem..

[18] Picard M., Shirihai O.S., Gentil B.J., Burelle Y. (2013). Mitochondrial morphology transitions and functions: Implications for retrograde signaling?. Am. J. Physiol. Regul. Integr. Comp. Physiol..

[19] Boland M.L., Chourasia A.H., Macleod K.F. (2013). Mitochondrial dysfunction in cancer. Front. Oncol..

[20] Jin P., Jiang J., Zhou L., Huang Z., Nice E.C., Huang C., Fu L. (2022). Mitochondrial adaptation in cancer drug resistance: Prevalence, mechanisms, and management. J. Hematol. Oncol..

[21] Rius-Pérez S., Torres-Cuevas I., Millán I., Ortega Á.L., Pérez S. (2020). PGC-1 *α*, inflammation, and oxidative stress: An integrative view in metabolism. Oxidative Med. Cell. Longev..

[22] Gao A., Jiang J., Xie F., Chen L. (2020). Bnip3 in mitophagy: Novel insights and potential therapeutic target for diseases of secondary mitochondrial dysfunction. Clin. Chim. Acta.

[23] Arfin S., Jha N.K., Jha S.K., Kesari K.K., Ruokolainen J., Roychoudhury S., Rathi B., Kumar D. (2021). Oxidative stress in cancer cell metabolism. Antioxidants.

[24] Muñoz-Sánchez J., Chánez-Cárdenas M.E. (2019). The use of cobalt chloride as a chemical hypoxia model. J. Appl. Toxicol..

[25] McDonald P.C., Dedhar S., Frost S.C., McKenna R. (2014). Carbonic Anhydrase IX (CAIX) as a Mediator of Hypoxia-Induced Stress Response in Cancer Cells. Carbonic Anhydrase: Mechanism, Regulation, Links to Disease, and Industrial Applications.

[26] Rayamajhi N., Kim S.-K., Go H., Joe Y., Callaway Z., Kang J.-G., Ryter S.W., Chung H.T. (2013). Quercetin induces mitochondrial biogenesis through activation of HO-1 in HepG2 cells. Oxidative Med. Cell. Longev..

[27] Grasso D., Zampieri L.X., Capelôa T., Van de Velde J.A., Sonveaux P. (2020). Mitochondria in cancer. Cell Stress.

[28] Chatterjee A., Dasgupta S., Sidransky D. (2011). Mitochondrial subversion in cancer. Cancer Prev. Res..

[29] Zhou J., Li G., Zheng Y., Shen H.-M., Hu X., Ming Q.-L., Huang C., Li P., Gao N. (2015). A novel autophagy/mitophagy inhibitor liensinine sensitizes breast cancer cells to chemotherapy through DNM1L-mediated mitochondrial fission. Autophagy.

[30] Aung L.H.H., Li R., Prabhakar B.S., Maker A.V., Li P. (2017). Mitochondrial protein 18 (MTP18) plays a pro-apoptotic role in chemotherapy—Induced gastric cancer cell apoptosis. Oncotarget.

[31] Locatelli L., Cazzaniga A., Fedele G., Zocchi M., Scrimieri R., Moscheni C., Castiglioni S., Maier J.A. (2021). A Comparison of doxorubicin-resistant colon cancer LoVo and leukemia HL60 cells: Common features, different underlying mechanisms. Curr. Issues Mol. Biol..

[32] Peoples J.N., Saraf A., Ghazal N., Pham T.T., Kwong J.Q. (2019). Mitochondrial dysfunction and oxidative stress in heart disease. Exp. Mol. Med..

[33] Richardson A.G., Schadt E.E. (2014). The role of macromolecular damage in aging and age-related disease. J. Gerontol. A Biol. Sci. Med. Sci..

[34] Detmer F.J., Alpert N.M., Moon S.-H., Dhaynaut M., Guerrero J.L., Guehl N.J., Xing F., Brugarolas P., Shoup T.M., Normandin M.D. (2022). PET imaging of mitochondrial function in acute doxorubicin-induced cardiotoxicity: A proof-of-principle study. Sci. Rep..

[35] Berthiaume J.M., Wallace K.B. (2007). Adriamycin-induced oxidative mitochondrial cardiotoxicity. Cell Biol. Toxicol..

[36] Osataphan N., Phrommintikul A., Chattipakorn S.C., Chattipakorn N. (2020). Effects of doxorubicin-induced cardiotoxicity on cardiac mitochondrial dynamics and mitochondrial function: Insights for future interventions. J. Cell. Mol. Med..

[37] Xie D., Wu X., Lan L., Shangguan F., Lin X., Chen F., Xu S., Zhang Y., Chen Z., Huang K. (2016). Downregulation of TFAM inhibits the tumorigenesis of non-small cell lung cancer by activating ROS-mediated JNK/P38MAPK signaling and reducing cellular bioenergetics. Oncotarget.

[38] Zhu Y., Xu J., Hu W., Wang F., Zhou Y., Xu W., Gong W., Shao L. (2020). TFAM depletion overcomes hepatocellular carcinoma resistance to doxorubicin and sorafenib through AMPK activation and mitochondrial dysfunction. Gene.

[39] Shen L., Sun B., Sheng J., Yu S., Li Y., Xu H., Su J., Sun L. (2018). PGC1α Promotes cisplatin resistance in human ovarian carcinoma cells through upregulation of mitochondrial biogenesis. Int. J. Oncol..

[40] Cortini M., Armirotti A., Columbaro M., Longo D.L., Di Pompo G., Cannas E., Maresca A., Errani C., Longhi A., Righi A. (2021). Exploring Metabolic adaptations to the acidic microenvironment of osteosarcoma cells unveils sphingosine 1-phosphate as a valuable therapeutic target. Cancers.

[41] Koshinaka K., Honda A., Masuda H., Sato A. (2020). Effect of quercetin treatment on mitochondrial biogenesis and exercise-induced AMP-activated protein kinase activation in rat skeletal muscle. Nutrients.

[42] McKay T.B., Lyon D., Sarker-Nag A., Priyadarsini S., Asara J.M., Karamichos D. (2015). Quercetin Attenuates lactate production and extracellular matrix secretion in keratoconus. Sci. Rep..

