# Supplementary material for: Contribution of Mitochondrial Activity to Doxorubicin-Resistance in Osteosarcoma Cells
Source: Cancers (Basel). 2023 Feb 21;15(5):1370. doi: 10.3390/cancers15051370 (PMC10000149; doi:10.3390/cancers15051370)
Supplement: Supplementary file 1 [file cancers-15-01370-s001.zip › cancers-2186459-supplementary.pdf]

Supplementary Materials: Contribution of Mitochondrial Activity to Doxorubicin-Resistance in Osteosarcoma Cells

Isabella Giacomini, Margherita Cortini, Mattia Tinazzi, Nicola Baldini, Veronica Cocetta, Eugenio Ragazzi, Sofia Avne and Monica Montopoli

Table S1. Cytotoxic effect of doxorubicin (calculated as IC_{50}) after 24, 48, and 72 hours of treatment in MG63, DXR 30 ng/mL, and MG63 DXR 100 ng/mL cell lines.

**Cell Line**	**IC_{50} Doxorubicin (ng/mL)[a]**		
	**24 Hours**	**48 Hours**	**72 Hours**
**MG63**	356.36 ± 5.11	143.68 ± 3.51	−3.23 ± 0.12
**MG63 DXR30 ng/mL**	1329.15 ± 9.12 ****	402.18 ± 4.22 ****	201.63 ± 7.40 ****
**MG63 DXR100 ng/mL**	820.54 ± 6.50 °°°°	485.95 ± 2.17 °°°°	308.38 ± 9.01 °°°°

[a] Values were determined by regression analysis and are expressed as mean ± SD of three different experiments. ****p<0.0001, MG63 DXR 30 ng/mL *vs* MG63 cells; °°°°p<0.0001, MG63 DXR 100 ng/mL vs. MG63 cells

Table S2. Cytotoxic effect of doxorubicin (calculated as IC_{50}) after 24, 48, and 72 hours of treatment in HOS, HOS DXR 10 ng/mL, HOS DXR 30 ng/mL, and HOS DXR 100 ng/mL cell lines.

**Cell Line**	**IC_{50} Doxorubicin (ng/mL)[a]**		
	**24 Hours**	**48 Hours**	**72 Hours**
**HOS**	180.36 ± 8.66	−96.67 ± 3.17	−144.89 ± 4.98
**HOS DXR10 ng/mL**	1550.42 ± 12.65 ****	331.15 ± 2.86 ****	362.64 ± 4.31 ****
**HOS DXR30 ng/mL**	1041.80 ± 1.50 °°°°	251.10 ± 2.35 °°°°	270.18 ± 1.61 °°°°

[a] Values were determined by regression analysis and are expressed as mean ± SD of three different experiments. ****p<0.0001, HOS DXR 10 ng/mL vs. HOS cells; °°°°p<0.0001, HOS DXR 30 ng/mL vs. HOS cells; ####p<0.0001, HOS DXR 100 ng/mL vs. HOS cells.

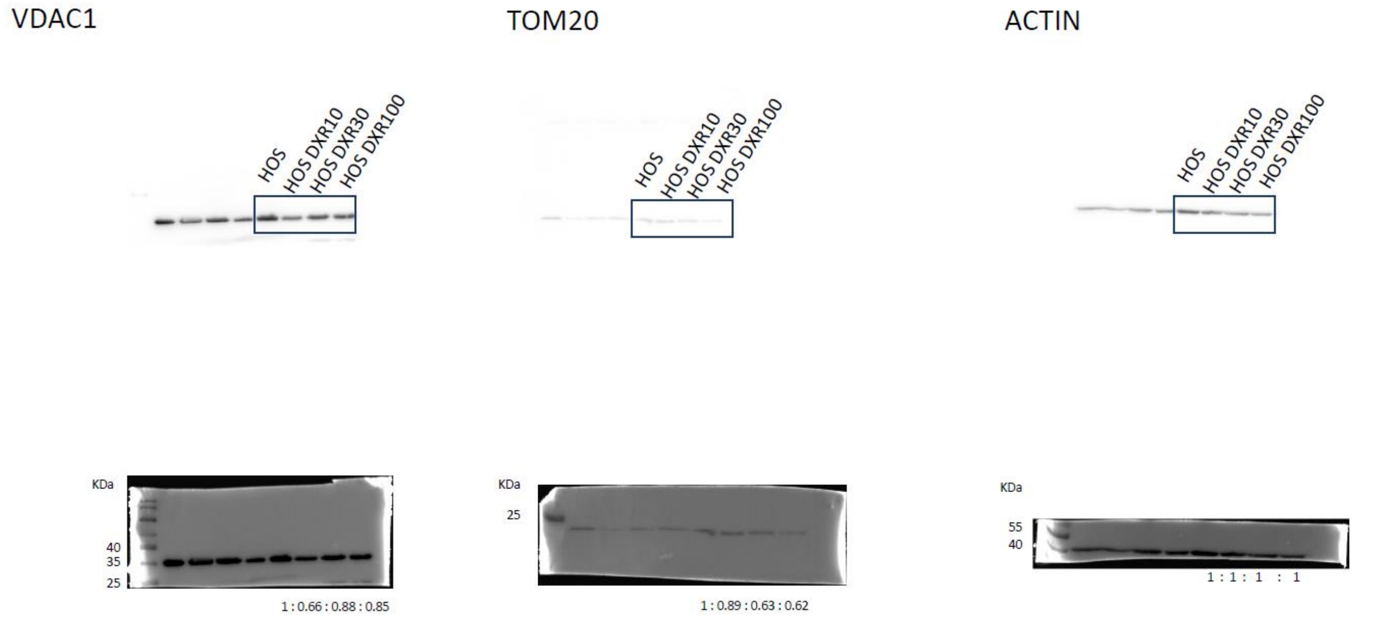

Figure S1. Western blot HOS.

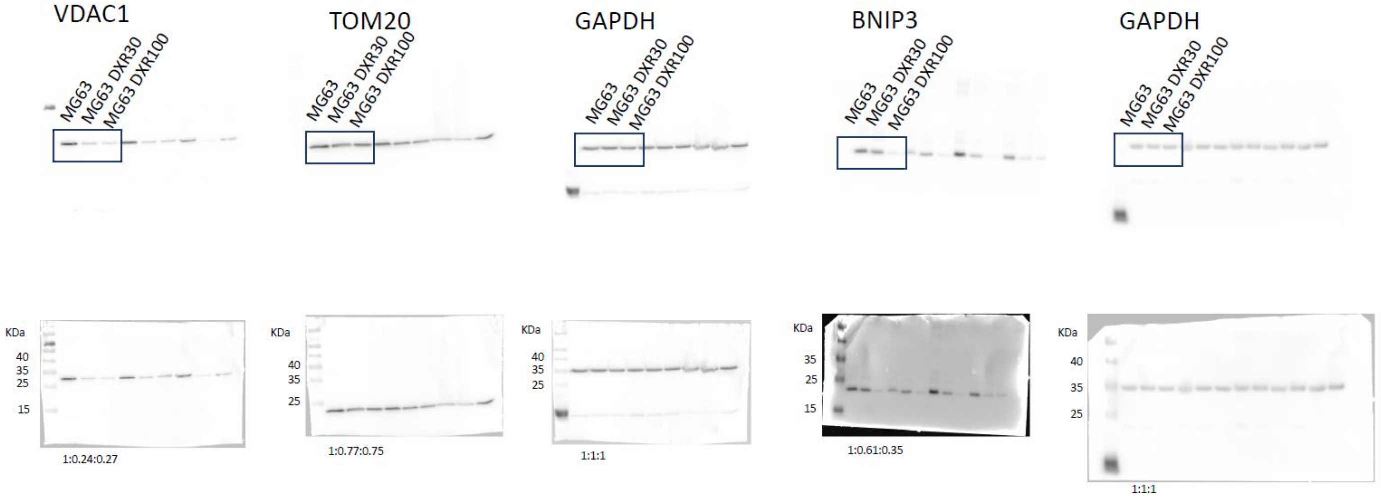

Figure S2. Western blot MG63.
